# Humic Acid Enhances Soil Fertility and Microbial Diversity Under Optimized Nitrogen Fertilization in Quinoa Rhizosphere

**DOI:** 10.3390/plants14243850

**Published:** 2025-12-17

**Authors:** Zeyun Guo, Jiaxing Gao, Tiantian Lv, Yan Zheng, Chenglei Deng, Xiaojing Sun, Yadi Sun, Chuangyun Wang, Yan Deng

**Affiliations:** College of Agriculture, Shanxi Agricultural University/Key Laboratory of Sustainable Dryland Agriculture of Shanxi Province, Taiyuan 030031, China; 20233124@stu.sxau.edu.cn (Z.G.); 202430134@stu.sxau.edu.cn (J.G.); lttlovelife@163.com (T.L.); 20233253@stu.sxau.edu.cn (Y.Z.); 202420105@stu.sxau.edu.cn (C.D.); z20223223@stu.sxau.edu.cn (X.S.); 20233269@stu.sxau.edu.cn (Y.S.); wcytyd@sxau.edu.cn (C.W.)

**Keywords:** quinoa, humic acid, nitrogen optimization, soil nutrient, rhizosphere microorganisms

## Abstract

Excessive nitrogen fertilization can degrade soil quality by inducing nutrient leaching, disrupting the microbial balance, and impairing plant reproductive growth, ultimately reducing crop yields. Optimizing nitrogen application rates and integrating humic acid fertilizers are promising strategies for enhancing soil fertility and improving agricultural productivity. The experimental design included four nitrogen application rates (N0:0 kg ha^−1^, N1:120 kg ha^−1^, N2:150 kg ha^−1^, and N3:180 kg ha^−1^) with and without humic acid (H: 1500 kg ha^−1^). Key findings revealed that: (1) The combined application of humic acid (1500 kg ha^−1^) and medium nitrogen (150 kg ha^−1^) significantly increased the contents of soil organic carbon (SOC), total nitrogen (TN), available phosphorus (AP), and available potassium (AK) by an average of 21.7% (*p* < 0.05), 90.5% (*p* < 0.01), 59.4% (*p* < 0.05), and 11.3% (*p* < 0.05), respectively (two-year mean), with significant interactive effects between nitrogen and humic acid on nutrient accumulation; (2) humic acid supplementation significantly increased soil bacterial abundance and diversity: under the combined treatment of medium nitrogen (150 kg ha^−1^) and humic acid, the bacterial Ace index (indicating species richness) and Shannon index (indicating community diversity) increased by an average of 0.76% and 0.30%, respectively, compared with the single medium nitrogen treatment (*p* < 0.05), promoting a more balanced microbial community; and (3) quinoa yields improved by 24.62–66.83% with humic acid application, with the highest yield increase observed under the moderate nitrogen rate (150 kg ha^−1^) in combination with humic acid. These results demonstrate that integrating humic acid with optimized nitrogen fertilization (150 kg ha^−1^ N + 1500 kg ha^−1^ HA) can effectively improve soil nutrients and enhance quinoa productivity. The increases in soil total nitrogen (TN, *p* < 0.01), available phosphorus (AP, *p* < 0.05), bacterial Shannon index (*p* < 0.05), and quinoa yield (*p* < 0.01) under this combined treatment were all significantly higher than those under single nitrogen fertilization or humic acid application, confirming the synergistic effect of the two fertilizers.

## 1. Introduction

Nitrogen is the primary limiting nutrient for plant growth and fundamentally determines the productivity and yield potential of crops [1]. Moreover, rational nitrogen application can significantly enhance crop biomass (including roots, straw, etc.). Crop residues such as stubble and fallen leaves returned to the soil after harvest provide the primary carbon source for soil organic matter (SOM), thereby promoting long-term increases in SOM content. Moderate nitrogen application also alleviates nitrogen limitation in soil microorganisms, promoting microbial fixation of organic carbon (e.g., forming microbial biomass carbon) and reducing mineralisation of organic carbon. This consequently increases the retention of soil organic matter [2], which is beneficial for improving soil productivity in the long term [3,4]. Nitrogen fertilizer is the largest source of nitrogen input in farmland, surpassing the typical contributions of biological nitrogen fixation, atmospheric deposition, and animal manure [5]. However, less than half of the nitrogen fertilizer usually applied to corn (*Zea mays* L.), rice (*Oryza sativa* L.) and wheat (*Triticum aestivum* L.) is actually absorbed by the crops, and the rest is stored in the soil or lost to the environment [6]. The loss of nitrogen in farmland has accelerated the depletion of soil alkaline cations, reduced regional water and air quality, exacerbated coastal hypoxia, and led to greenhouse gas emissions [7]. Globally, inefficient nitrogen use in agricultural systems exacerbates these issues. Lassaletta et al. found that only 47% of nitrogen inputs to global croplands are taken up by crops, with the remaining 53% lost to the environment through multiple pathways—including leaching, runoff, volatilization, and denitrification [8], Denitrification, in particular, contributes to substantial nitrogen loss in many agricultural soils by converting nitrate to gaseous nitrogen compounds (e.g., N_2_O, N_2_) under anaerobic conditions, a process that is prevalent even in dryland systems during episodic moisture events. This pattern of low nitrogen retention aligns with the low nitrogen use efficiency observed in major cereal crops within this study area, where denitrification further contributes to nitrogen losses in the dryland cropping systems investigated. The resulting environmental impact spreads through three main channels: (1) groundwater pollution caused by nitrate leaching, (2) surface water eutrophication caused by nitrogen transport mediated by runoff, and (3) air pollution caused by ammonia volatilization and the subsequent formation of particulate matter [9,10]. In recent decades, the excessive use of mineral fertilizers in conventional agriculture has caused irreplaceable damage to ecosystems, including soil pollution, disruption of the soil biological balance, and reduction in product quality [11]. Therefore, addressing these challenges is crucial for promoting sustainable agricultural systems and mitigating the ecological degradation associated with poor nitrogen management [12,13].

The adoption of high-efficiency fertilizers has become a cornerstone strategy for sustainable agriculture, offering dual benefits: reducing the environmental impact of excessive fertilization while maintaining optimal crop productivity [14]. The excessive use of mineral fertilizers has caused serious ecological problems, prompting interest in humic acid as a sustainable alternative in agriculture [15]. 

Humic substances (HS) have emerged as a natural alternative to traditional agricultural practices that rely excessively on mineral fertilizers to maintain soil fertility and crop yield, specifically by replacing the overuse of inorganic nitrogen, phosphorus, and potassium fertilizers that lead to soil acidification, microbial imbalance, and nutrient leaching. Per definition, humic substances encompass extractable humic acids and fulvic acids [16]. They are organic substances produced and accumulated by the decomposition and transformation of animal and plant remains via microorganisms and a series of geochemical processes. The combined application of humic substances and various inorganic fertilizers can improve soil quality, increase the fertilizer utilization rate [17,18], and enhance crop yield and quality [19,20,21]. From an agricultural perspective, humic acid has long been regarded as a valuable organic conditioner. They have been widely used over the past few decades to promote crop growth and yields [22]. Humic acid improve soil structure by decomposing clay, buffering pH values, enhancing water retention, and increasing the availability of soil nutrients [23,24]. They enhance soil fertility by promoting the release of trace elements and nutrients, stimulating the physiological development of plants, and increasing their tolerance to environmental stress [25,26]. Scientific research indicates that excessive fertilization, especially the intensive application of chemical fertilizers, can significantly reduce soil bacterial diversity and potentially disrupt the balance of microbial communities and ecological functions [27]. Humic acid can stimulate the activity of soil microorganisms [28]. By selectively promoting bacterial communities, such as Acidobacterium and actinomycetes [23], it significantly alters the population dynamics of soil microorganisms, affecting community diversity and exerting a profound impact on the soil microbial ecosystem [29,30,31]. Studying the effect of humic acid on soil microbial communities offers crucial insights into its potential to improve soil quality. Microorganisms, as the most biologically active components of soil ecosystems, are involved in fundamental biochemical processes on Earth, including the mineralisation of organic matter, humification, and the transformation and cycling of nutrients [32,33]. These synergistic effects create favorable conditions for root growth, ultimately increasing crop productivity [34].

This study simultaneously applied nitrogen fertilizer and humic acid based on their synergistic enhancement mechanism of quinoa growth. It focused on examining the effects of humic acid on the quinoa rhizosphere ecosystem, with particular emphasis on soil nutrient dynamics, microbial activity characteristics, yield performance, and community composition. This approach addresses the core nutritional requirements of quinoa while overcoming the limitations of single-fertilizer applications. We hypothesized that: (1) for key parameters including aboveground dry matter accumulation of quinoa, soil nutrient contents (SOC, TN, AP, AK), and bacterial diversity indices (Ace, Shannon), humic acid (HA) addition would exert a significant positive effect regardless of nitrogen (N) application level; (2) there would be significant interactive effects between N levels (0, 120, 150, 180 kg ha^−1^) and HA on the above parameters—specifically, HA would enhance the responses of soil nutrients and bacterial diversity to medium N (150 kg ha^−1^) more strongly than to low N (120 kg ha^−1^) or high N (180 kg ha^−1^), and this synergy would further promote dry matter accumulation and yield formation of quinoa.

## 2. Materials and Methods

### 2.1. Test Site

Field trials were conducted at a quinoa cultivation base in Niangzhen Township, Jingle County, Xinzhou City, Shanxi Province (elevation 1140–2421 m, 38.17° N, 112.1° E). This region has a temperate monsoon climate. This area has four distinct seasons. It is warm in summer, and the temperature between day and night varies significantly. Meteorological records indicate that the annual sunshine duration is 2450 h and the frost-free period ranges from 120 to 135 days. The precipitation was 610.2 mm (in 2023) and 506.8 mm (in 2024), and the average temperatures were 16.3 °C and 8.4 °C, respectively. Soil samples from the 0–20 cm soil layer were analyzed, and the soil was classified as Calcaric Regosol, according to the World Reference Base for Soil Resources (WRB) classification system. The measurements in 2023 revealed the following soil characteristics: SOM, 7.87 g kg^−1^; TN, 0.95 g kg^−1^; AP, 21.31 mg kg^−1^; AK, 132 mg kg^−1^; and pH, 8.09. The soil parameters determined in 2024 were as follows: SOM, 7.56 g kg^−1^; TN, 0.87 g kg^−1^; AP, 17.52 mg kg^−1^; AK, 126.02 mg kg^−1^; and pH, 8.20.

### 2.2. Experimental Design

This study employed a two-factor split-plot design. The specific treatment details are presented in Table 1. In the experimental design, nitrogen fertilizer was considered the main factor (0, 120, 150, and 180 kg ha^−1^, referred to as N0–N3), and humic acid was considered the secondary factor (1500 kg ha^−1^, referred to as H). Eight different treatments were established. Each treatment was repeated three times on a 50 m^2^ (5 m × 10 m) plot. The test plan begins on May 27th every year and adopts a mechanical precision sowing. During the land preparation process, apply urea and organic fertilizer rich in humic acid evenly as a base fertilizer. Humic acid fertilizer was obtained from Xinhe Biotechnology Co., Ltd. in Shandong Province, China. All treatments were carried out using standardized field management methods, and the application amounts of phosphorus and K fertilizers were determined. The growth parameters of quinoa were systematically monitored throughout the growing season after fertilization.

### 2.3. Field Management

The primary raw material for this humic acid fertilizer is micro-activated weathered coal, which is mechanically milled into a fine powder. The formulation utilizes a commercially sourced, purified humic acid (purity ≥ 90%). The product contains ≤10% filler by mass, consisting of inert components that have no impact on experimental outcomes. Its nutritional components are shown in Table 2. No mineral additives (such as urea or superphosphate) were added during production, confirming its organic nature. All treatments were performed using standardized field management methods. Phosphorus fertilizer (P content 5.24%) at 80 kg ha^−1^ and potassium fertilizer (K content 41.5%) at 60 kg ha^−1^ were uniformly applied at one time before sowing in combination with land preparation. No other fertilizers were used throughout the entire growth period to avoid additional nutrients interfering with the test results. Weed control is carried out manually once during the branching stage and once during the flowering stage. This manual weeding method avoids the use of chemical herbicides (which may directly inhibit soil microbial activity). It eliminates potential adverse effects of weeds on soil microorganisms: (1) Preventing allelopathic effects—weeds (e.g., Amaranthus retroflexus and Setaria viridis common in the experimental area) release allelochemicals (e.g., phenolic acids, terpenoids) that can suppress the growth and metabolic activity of rhizosphere beneficial microorganisms (e.g., Proteobacteria and Actinobacteria); (2) Reducing resource competition—weeds compete with quinoa for soil nutrients (N, P, K) and carbon sources, and their removal ensures sufficient nutrient supply for soil microorganisms, avoiding microbial community imbalance caused by nutrient deficiency. No chemical pesticides were used throughout the growth period, avoiding interference with soil microorganisms and crop quality. The growth parameters of quinoa were systematically monitored throughout the growing season after fertilization.

### 2.4. Measurement Method

#### 2.4.1. Soil Sample Collection, Preparation and Nutrient Content Detection

Before sowing (May 25), a standardized three-point sampling method with a depth of 0–20 cm was employed. During the physiological maturity period (October 10th), five representative quinoa plants with intact rhizosphere were randomly selected from each experimental plot. All samples were immediately transported to the laboratory in sterile, breathable, self-sealing bags under controlled temperature conditions. Laboratory processing included: (1) rapid freezing of sub-samples at −80 °C for subsequent microbial community analysis; (2) the remaining samples after air-drying and sieving (60 mesh, 0.246 mm aperture) were used for physical and chemical analysis, including pH determination and nutrient quantification. The physical and chemical properties of the soil were determined using the agricultural chemical analysis method. The pH value was measured using a pH meter (PHS-3C, Shanghai, China). SOM was determined using the potassium dichromate oxidation method, and SOC was calculated. TN was determined using the Kjeldahl nitrogen determination method and digestion with molybdenum-antimony reverse-phase spectrophotometry. AP was determined using the molybdenum blue method. AK was determined using a 1 mol L^−1^ ammonium acetate (NH_4_OAc) solution buffered to pH 7.0 for extraction, followed by detection with a flame photometer. This method conforms to the national standard of China (NY/T 889-2017 Soil Testing Method for Available Potassium in Farmland Soil) and international standard protocols for soil available nutrient analysis.

#### 2.4.2. Sequencing

##### Extraction of the Genome DNA

Total genomic DNA was extracted from the samples using the CTAB/SDS method. DNA concentration and purity were monitored on 1% agarose gels. According to the concentration, the DNA was diluted to 1 ng/μL using sterile water.

##### Amplicon Generation

Primer: 16S V4-V5: 515F-907R, 18S V9: 1380F-1510R, ITS1: ITS1F-ITS2R. 16S/18S rRNA genes were amplified using specific primers with barcodes. All PCR reactions were carried out in 30 μL reactions with 15 μL of Phusion^®^High-Fidelity PCR Master Mix (New England Biolabs, MA, USA), 0.2 μM of forward and reverse primers, and approximately 10 ng template DNA. Thermal cycling consisted of initial denaturation at 98 °C for 1 min, followed by 30 cycles of denaturation at 98 °C for 10 s, annealing at 50 °C for 30 s, and elongation at 72 °C for 60 s. Finally, 72 °C for 5 min.

##### PCR Products Quantification and Qualification

The same volume of 1X loading buffer (containing SYBR green) was mixed with the PCR products, and electrophoresis was performed on a 2% agarose gel for detection. Samples with a bright main strip between 400 and 450 bp were chosen for further experiments.

##### PCR Products Mixing and Purification

The PCR products were mixed in equidensity ratios. The mixture of PCR products was purified using the GeneJET Gel Extraction Kit (Thermo Scientific).

##### Library Preparation and Sequencing

Sequencing libraries were generated using the NEBNext Ultra DNA Library Prep Kit for Illumina (NEB, USA) according to the manufacturer’s recommendations, and index codes were added. Library quality was assessed using a Qubit@ 2.0 Fluorometer (Thermo Scientific) and an Agilent Bioanalyzer 2100 system. Finally, the library was sequenced on an Illumina MiSeq platform, and 250 bp/300 bp paired-end reads were generated.

#### 2.4.3. Composition and Yield of Quinoa Production

During the maturity period of quinoa, 10 uniformly growing plants were randomly selected from each plot, and the number of branches and the length of the central spike were recorded. The quinoa ears were placed in net bags, threshed, and dried in the sun. Subsequently, a JM-A 20002 electronic balance was used for weighing. This seed sample was used to determine the yield and thousand-grain weights.

### 2.5. Data Analysis

The data collected in this study were processed using Microsoft Excel 2019 (Microsoft, Redmond, WA, USA), and the significance of the data was analyzed using the DPS data processing system. SPSS 27.0 statistical analysis software was used for analysis of variance and correlation analysis. The chart was created using Origin 2021 software. Species richness and diversity were evaluated using the α-diversity indices (Chao, Ace, Shannon) and the β-diversity index (PCoA). The correlations among the microbial communities were explored using redundancy analysis (RDA). We adopted the natural logarithmic correspondence ratio (lnRR) [35] as a measure of the response of soil microbial communities to soil nutrients, and the lnRR effect size was calculated as follows:

$$\ln RR=\ln(\frac{{\bar{X}}_{t}+S_{t}\;}{{\bar{X}}_{c}+S_{c}})=\ln({\bar{X}}_{t}+S_{t})-\ln({\bar{X}}_{c}+S_{c})$$

Among them, (${\bar{X}}_{t}$) and (${\bar{X}}_{c}$) are the average values of the experimental group and the reference point (control group), respectively. ($S_{t}$) and ($S_{c}$) represent the sample standard deviation of the experimental group and the sample standard deviation of the mean value of the reference point (control group), respectively. A linear regression analysis was conducted to determine the relationship between soil microbial communities and soil nutrients.

## 3. Results

### 3.1. Production Analysis

**Figure 1:**
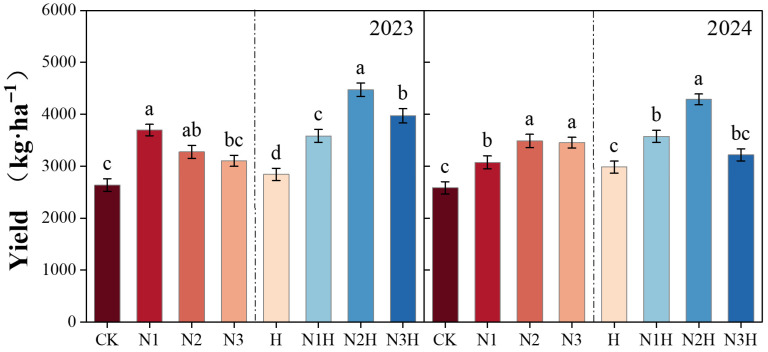
The impact of different fertilization treatments on yield from 2023 to 2024. CK (0 kg ha^−1^ nitrogen fertilizer); N1 (120 kg ha^−1^ nitrogen fertilizer); N2 (150 kg ha^−1^ nitrogen fertilizer); N3 (180 kg ha^−1^ nitrogen fertilizer); H (1500 kg ha^−1^ humic acid); N1H (120 kg ha^−1^ nitrogen fertilizer and 1500 kg ha^−1^ humic acid); N2H (150 kg ha^−1^ nitrogen fertilizer and 1500 kg ha^−1^ humic acid); and N3H (180 kg ha^−1^ nitrogen fertilizer and 1500 kg ha^−1^ humic acid). Different letters on the bar chart denote statistically significant differences (*p* < 0.05) identified by Duncan’s multiple range test.

Humic acid application increased the yield indicators of quinoa, including the number of branches, thousand-grain weight, grain weight per plant, and main spike length. Over the two-year trial, the yield increases from humic acid treatment exceeded those from nitrogen fertilizer alone, with significant differences (7.31–66.83% in 2023 and 15.51–66.03% in 2024) (*p* < 0.05; Figure 1). In the single nitrogen fertilizer application treatment, increases were observed in the number of branches (9.43–55.17%), 1000-grain weight (14.80–55.40%), single-plant grain weight (0.59–31.07%), and main spike length (17.22–40.20%) compared to the control treatment. After humic acid application, the increases in these yield indicators were significantly greater than those from nitrogen fertilizer alone, with the number of branches increasing by 3.77–71.43%, 1000-grain weight by 10.18–79.94%, single-plant grain weight by 7.40–39.32%, and main spike length by 7.88–69.65% (Table 3), respectively.

### 3.2. Effects of Different Nitrogen Fertilizers Combined with Humic Acid on Soil Nutrient Content

**Figure 2:**
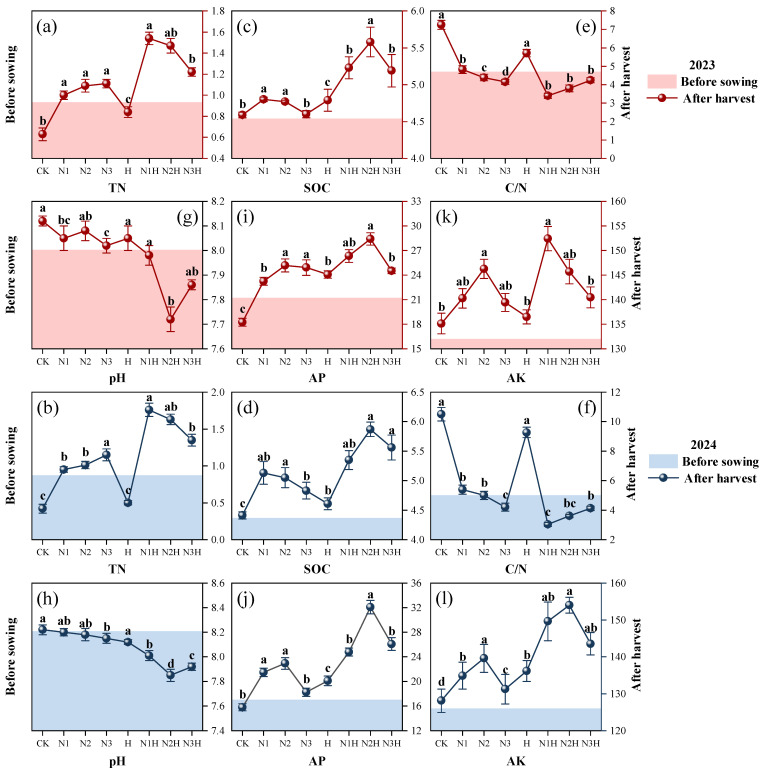
The effects of different fertilization treatments on soil total nitrogen (TN), soil organic carbon (SOC), carbon-to-nitrogen ratio (C/N), pH, available phosphorus (AP), and available potassium (AK) from 2023 to 2024 were evaluated. CK (0 kg ha^−1^ nitrogen fertilizer), N1 (120 kg ha^−1^ nitrogen fertilizer), N2 (150 kg ha^−1^ nitrogen fertilizer), N3 (180 kg ha^−1^ nitrogen fertilizer), H (1500 kg ha^−1^ humic acid), N1H (120 kg ha^−1^ nitrogen fertilizer and 1500 kg ha^−1^ humic acid), N2H (150 kg ha^−1^ nitrogen fertilizer and 1500 kg ha^−1^ humic acid), and, N3H (180 kg ha^−1^ nitrogen fertilizer and 1500 kg ha^−1^ humic acid). (**a**) 2023 TN; (**b**) 2024 TN; (**c**) 2023 SOC; (**d**) 2024 SOC; (**e**) 2023 C/N; (**f**) 2024 C/N; (**g**) 2023 pH; (**h**) 2024 pH; (**i**) 2023 AP; (**j**) 2024 AP; (**k**) 2023 AK; (**l**) 2024 AK; Different letters on the bar chart denote statistically significant differences (*p* < 0.05) identified by Duncan’s multiple range test.

Nitrogen application levels and humic acid had significant effects on soil nutrient content (Tables S1 and S2). After the addition of humic acid, the TN, AP, AK, and SOC contents in the soil increased significantly (R = 0.90, *p* < 0.05) (Figure 2). Among them, the TN content ranged from 3.21 to 4.19 times that of the control treatment (Figure 2a,b). With the nitrogen application rate increasing from 120 kg ha^−1^ to 180 kg ha^−1^, the soil SOC content first increased and then decreased. When the dosage of humic acid was 150 kg ha^−1^, the SOC content in the soil peaked (Figure 2c,d). The soil pH results showed that in 2023, the pH value of the group applying nitrogen fertilizer alone decreased by 0.12% to 0.84%, while that of the group adding humic acid decreased by 0.49% to 4.51%. The pH levels in 2024 were consistent with those in 2023. After the addition of humic acid, the pH decreased by 2.32% to 4.27% (Figure 2g,h). Under the condition of applying nitrogen fertilizer alone, the AP content in the soil reached its peak when the nitrogen application rate was 150 kg ha^−1^. After humic acid application, the trend in soil AP content was consistent with that of the nitrogen fertilizer application alone. However, the AP content was significantly higher than that of N-fertilizer application alone (Figure 2i,j). After the application of humic acid, the AK content in the soil increased by 9.37% to 10.91% compared to the application of nitrogen fertilizer alone (Figure 2k,l).

### 3.3. Effects of HA Application on Rhizosphere Soil Microorganisms

#### 3.3.1. Relative Abundance of Major Bacterial Phyla

**Figure 3:**
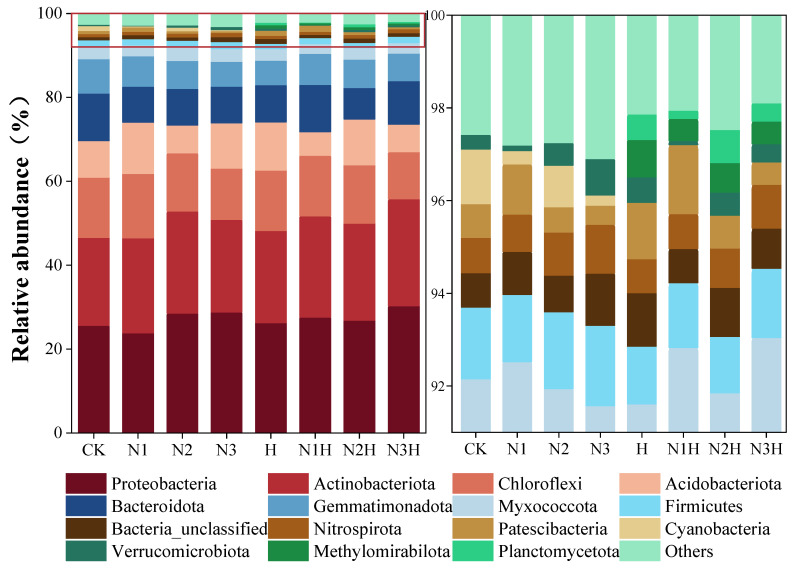
Soil bacterial community phylum levels under different fertilization treatments. CK (0 kg ha^−1^ nitrogen fertilizer); N1 (120 kg ha^−1^ nitrogen fertilizer); N2 (150 kg ha^−1^ nitrogen fertilizer); N3 (180 kg ha^−1^ nitrogen fertilizer); H (1500 kg ha^−1^ humic acid); N1H (120 kg ha^−1^ nitrogen fertilizer and 1500 kg ha^−1^ humic acid); N2H (150 kg ha^−1^ nitrogen fertilizer and 1500 kg ha^−1^ humic acid); and N3H (180 kg ha^−1^ nitrogen fertilizer and 1500 kg ha^−1^ humic acid). The enlarged area is indicated by the red box on the right-hand side of the image.

Analysis of treatment on OTUs indicated that fertilization significantly affected the composition of soil bacterial communities during the quinoa maturation period. Fifteen dominant bacterial phyla were identified. The most numerous were Proteobacteria (23.68–30.32%), Actinomycetes (20.83–25.48%), and Campylobacter (11.22–15.23%), accounting for 64.05% of the total sequence. Other subdominant groups included Acidobacteria (5.58–12.18%), Bacteroides (7.52–11.29%), and Bacillus (5.79–8.15%). The secondary components included the Myxococcus phylum, the Thick-wall bacteria phylum, and the Nitrifying Screw phylum, each of which accounted for less than 3.2% of the sequence (Figure 3). In the N1, N2, and N3 treatments, Proteobacteria and Actinomycetes were less abundant than in the humic acid treatments (N1H, N2H, N3H), whereas Campylobacter abundance was higher. The combined application of nitrogen and humic acid reduced the Campylobacter phylum by 11.22–14.49%, compared to 12.10–15.23% with nitrogen alone. Bacteroidetes abundance was lower in all fertilized treatments than in CK, with nitrogen alone having the lowest abundance. Bacillimonas phylum remained stable (5.79–8.21%) across treatments. Myxomycota and Nitrospira were more abundant with nitrogen alone, while Floxomycota and methanotrophic bacteria were only found in combined treatments. Cyanobacteria (0.22–1.17%) were only present in the nitrogen-only treatments.

#### 3.3.2. Diversity of Bacterial Communities

**Figure 4:**
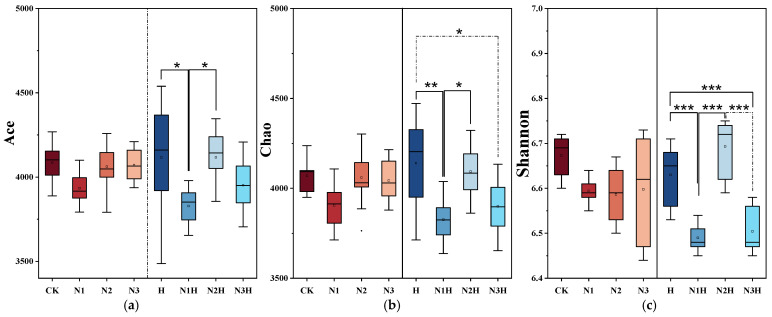
Alpha diversity analysis of bacterial communities under different fertilization treatments. CK (0 kg ha^−1^ nitrogen fertilizer); N1 (120 kg ha^−1^ nitrogen fertilizer); N2 (150 kg ha^−1^ nitrogen fertilizer); N3 (180 kg ha^−1^ nitrogen fertilizer); H (1500 kg ha^−1^ humic acid); N1H (120 kg ha^−1^ nitrogen fertilizer and 1500 kg ha^−1^ humic acid); N2H (150 kg ha^−1^ nitrogen fertilizer and 1500 kg ha^−1^ humic acid); and N3H (180 kg ha^−1^ nitrogen fertilizer and 1500 kg ha^−1^ humic acid). * indicate significance at the 0.05 level, ** indicate significance at the 0.01 level, and *** indicates extreme significance at the 0.001 significance level. (**a**) Ace Index; (**b**) Chao Index; (**c**) Shannon Index.

Microbial diversity was assessed using alpha diversity indices, which quantify species abundance and distribution within the individual samples. Operational Taxonomic Units (OTUs) indicate the total number of annotated sequences. Ace and Chao indices measure species richness, while the Shannon index evaluates overall diversity and evenness [36]. In a series of fertilization treatment experiments, the Ace index (Figure 4a), Chao index (Figure 4b), and Shannon index (Figure 4c) were used to study the effects of different treatments on the bacterial diversity of the rhizosphere soil of quinoa. Compared with CK, the Ace and Chao indices of N1H and N3H treatments decreased (Ace: 3.28–6.30%; Chao: 4.19–6.01%), while those of N2H slightly increased (Ace: 0.76%; Chao: 0.58%), and H treatment increased (Ace: 0.76%; Chao: 1.72%). Different nitrogen-decomposition co-application treatments significantly influenced the α-diversity of bacteria in the quinoa rhizosphere soil. Distinct variations in the Shannon index clearly reflected the synergistic regulation between species richness and evenness. In terms of index characteristics, the Ace index directly measures species richness (the total number of species in the community). The Shannon index integrates both richness and evenness (the balanced distribution of species across taxonomic units). Specifically, the N2H treatment (medium nitrogen + humic acid) showed a 0.76% increase in the Ace index compared to CK, directly demonstrating a significant increase in the species richness. The Shannon index increased by 0.30 simultaneously. Combined with the change in the Ace index, this improvement reflects the combined effect of “increased richness + optimized evenness.” While dominant groups (e.g., Proteobacteria and Actinobacteria) showed increased abundance, no single species achieved excessive dominance. Functional bacteria with medium-to-low abundance (e.g., Bacillobacteria involved in phosphorus cycling) also proliferated effectively, consistent with the “synergistic growth of multi-phylum microbial communities” observed in Figure 2. The high-N + humic acid (N3H) treatment exhibited opposite trends: the Ace index decreased by 0.42% compared to the N2H treatment, and the Shannon index declined by 0.21%. This indicates that excessive nitrogen application suppresses species richness while also disrupting community evenness. Under high-N conditions, a few dominant nitrogen-tolerant bacteria (e.g., Pseudomonas) proliferated rapidly, crowding out the ecological niches of other functional bacteria and resulting in an imbalanced species distribution. This observation further explains why the soil nutrient enhancement effect was less pronounced in the N3H treatment than in the N2H treatment group.

**Figure 5:**
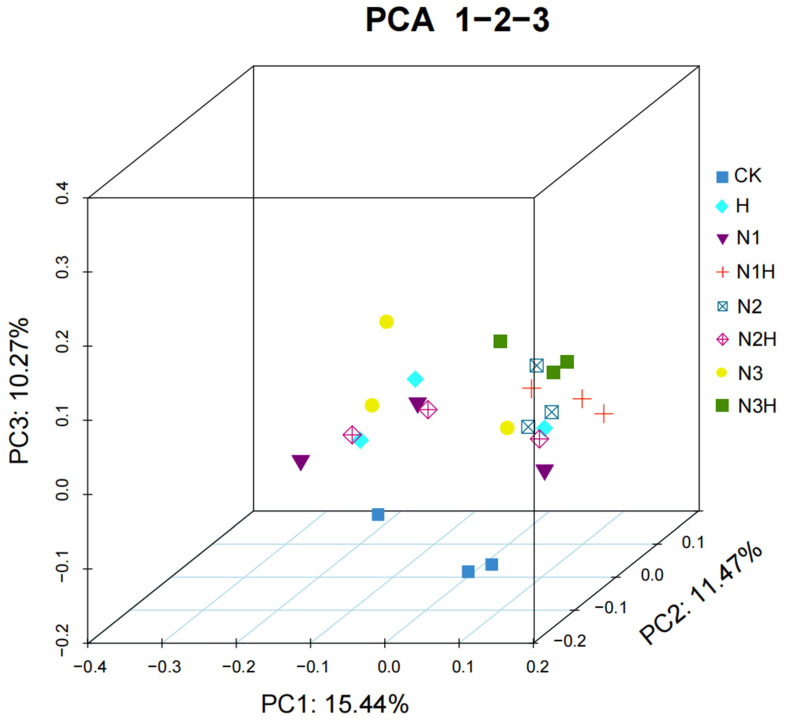
Soil bacteria (OUT level) community PCoA analysis. CK (0 kg ha^−1^ nitrogen fertilizer); N1 (120 kg ha^−1^ nitrogen fertilizer); N2 (150 kg ha^−1^ nitrogen fertilizer); N3 (180 kg ha^−1^ nitrogen fertilizer); H (1500 kg ha^−1^ humic acid); N1H (120 kg ha^−1^ nitrogen fertilizer and 1500 kg ha^−1^ humic acid); N2H (150 kg ha^−1^ nitrogen fertilizer and 1500 kg ha^−1^ humic acid); and N3H (180 kg ha^−1^ nitrogen fertilizer and 1500 kg ha^−1^ humic acid).

No significant differences were observed between nitrogen fertilizer application alone and CK for any of the indices. Microbial β-diversity was evaluated using PCoA based on the Bray–Curtis distance, and the results are presented as a three-dimensional scatter plot (Figure 5). Microbial β-diversity was evaluated using PCoA based on the Bray–Curtis distance, and the results were presented as a three-dimensional scatter plot.

### 3.4. Correlation Analysis

#### 3.4.1. The Relationship Between Soil Nutrient Composition and Microbial Communities

**Figure 6:**
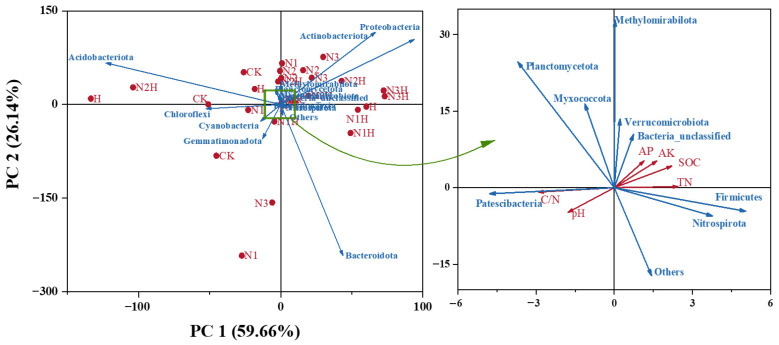
Redundancy Analysis of Soil Physical and Chemical Indicators and Soil Bacterial Communities. CK (0 kg ha^−1^ nitrogen fertilizer); N1 (120 kg ha^−1^ nitrogen fertilizer); N2 (150 kg ha^−1^ nitrogen fertilizer); N3 (180 kg ha^−1^ nitrogen fertilizer); H (1500 kg ha^−1^ humic acid); N1H (120 kg ha^−1^ nitrogen fertilizer and 1500 kg ha^−1^ humic acid); N2H (150 kg ha^−1^ nitrogen fertilizer and 1500 kg ha^−1^ humic acid); N3H (180 kg ha^−1^ nitrogen fertilizer and 1500 kg ha^−1^ humic acid); TN (Total Nitrogen); SOC (Soil Organic Carbon); C/N (Carbon to Nitrogen Ratio); pH (Acidity or Alkalinity); AP (Available Phosphorus); AK (Available Potassium).

**Figure 7:**
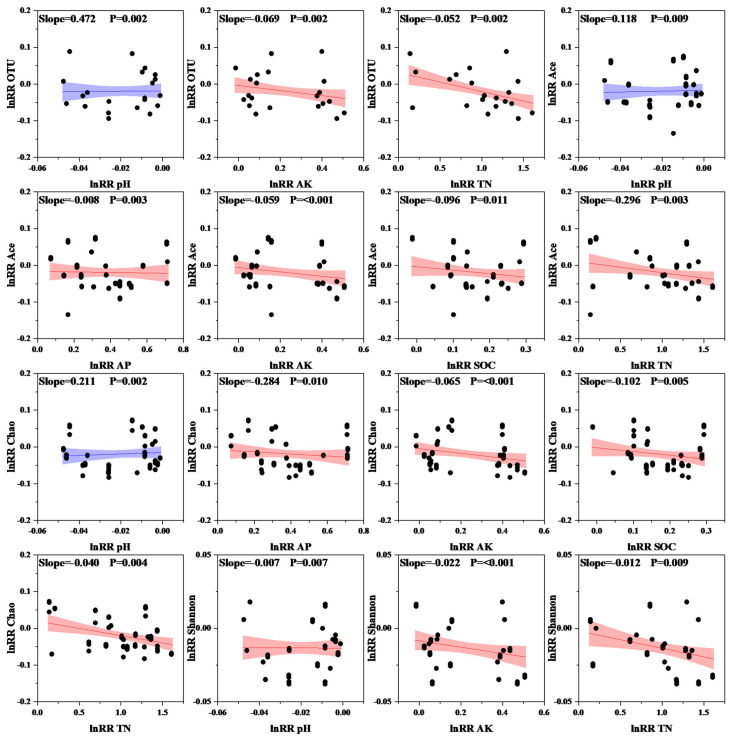
Relationship between soil microbial diversity and environmental factors; blue color indicates a positive correlation, and red color indicates a negative correlation. The shaded areas correspond to the 95% confidence interval. TN: total nitrogen; SOC: organic carbon; AP: available phosphorus; AK: available potassium.

To further analyze the relationship between soil nutrient composition and microbial communities, we conducted a redundancy analysis (RDA). The results showed that PC1 and PC2, the first and second principal components, respectively, had contribution rates of 59.66% and 26.14% (Figure 6), accounting for 85.80% of the variation in the bacterial community. Proteobacteria, Actinomycetes, methanotrophic bacteria, Unclassified Bacteria, Nitrifying Helicobacter, Verrucomicrobia, and Firmicutes were positively correlated with AP, AK, TN, and SOC but negatively correlated with pH and C/N. In contrast, Planctomycetes, Bacteroidetes, Acidobacteria, Campylobacterota, Bacillimonas, and Cyanobacteria were negatively correlated with AP, AK, TN, and SOC and positively correlated with pH and C/N. Bacterial OTUs, Ace, and Chao indices exhibited extremely significant positive correlations with increasing pH values. The bacterial Ace and Chao indices were significantly negatively correlated with increasing AP, AK, SOC, and TN (Figure 7). The relationships between soil microbial community responses and environmental factors, as well as soil physicochemical properties, are listed in Table 4 (only significant relationships).

#### 3.4.2. Relationship Between Soil Nutrient Composition, Yield and Microbial Community

**Figure 8:**
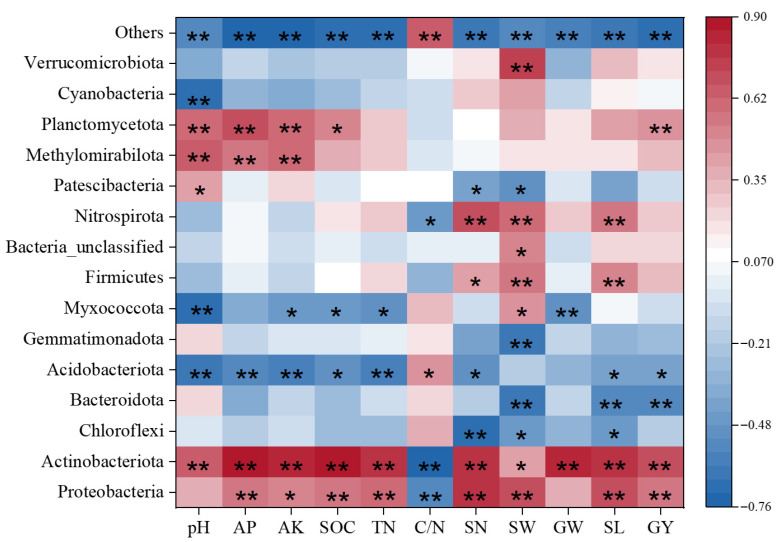
Correlation heatmap of bacterial abundance with soil nutrients and quinoa yield. TN (Total Nitrogen); SOC (Soil Organic Carbon); C/N (carbon-to-nitrogen ratio); pH (Acidity or Alkalinity); AP (Available Phosphorus); AK (Available Potassium); SN (number of spikes per plant); SW (1000-grain weight); GW (grain weight per plant); SL (main spike length); GY (Yield). * indicate significance at the 0.05 level, ** indicate significance at the 0.01 level.

The abundance of Proteobacteria and Actinomycetes was positively correlated with pH, AP, AK, TN, SOC, and yield, as well as yield components, but negatively correlated with the carbon-to-nitrogen ratio. Conversely, Campylobacterota, Acidobacteria, Bacteroidetes, and Bacillimonas were negatively correlated with AP, AK, TN, SOC, and yield, as well as yield components, and positively correlated with the carbon-to-nitrogen ratio. The phyla Firmicutes and Nitrospira were positively correlated with yield and yield components but negatively correlated with pH, AP, and AK. The phyla Methanotrophic bacteria and Fusarium were significantly positively correlated with pH, AP, and AK (Figure 8).

## 4. Discussion

### 4.1. Effects of Nitrogen Fertilizer Combined with Humic Acid on Soil Properties of Quinoa

The study revealed that nitrogen fertilizer application was significantly positively correlated with soil organic carbon (SOC) content, which increased with higher nitrogen inputs. However, at high nitrogen levels (180 kg ha^−1^), the SOC enhancement rate was significantly lower than that at medium nitrogen levels (120 kg ha^−1^). This could be because nitrogen fertilizers stimulate higher biomass input from quinoa, increasing litter and root exudates, and thus enhancing soil carbon input [37]. Notably, when nitrogen application rates exceeded 180 kg ha^−1^, excessive nitrogen input significantly stimulated the activity of decomposer microorganisms (e.g., saprophytic bacteria and actinomycetes) in the soil, accelerating the mineralization of soil organic matter. At this stage, the decomposition rate of organic matter exceeds the carbon input rate from nitrogen-enhanced sources (such as litter and root exudates), thereby preventing any net increase in SOC content and potentially leading to a decrease. This is consistent with Geisseler and Scow, who found that excessive nitrogen input alters soil microbial community structure, specifically increasing the abundance and activity of functional microorganisms involved in decomposition, thereby enhancing the mineralization of soil organic carbon [38]. Nitrogen application reduces soil respiration and CO_2_ emissions while increasing the soil SOC content [39]. Humic acid, an external carbon source, significantly boosts soil microbial abundance and activity, enhances carbon sequestration, reduces greenhouse gas emissions, and plays a key role in increasing soil SOC [40]. The carbon-to-nitrogen ratio, as a key indicator of the dynamic decomposition of organic carbon, further validates these findings. Previous studies have found that long-term application of inorganic or organic fertilizers can significantly reduce soil pH [41], which is consistent with the results of this experiment (Figure 2g,h). Humic acid has a strong acid-base buffering capacity, reducing salinization and regulating soil pH towards neutrality. Yue Yuan found that humic acid significantly improves phosphorus availability in calcareous soils and nutrient utilization rates by altering phosphorus forms and reducing fixation, outperforming traditional nitrogen fertilizers [42]. This study also confirmed that the application of humic acid can significantly increase AP and AK in quinoa during its maturation period, verifying the above results.

### 4.2. Effects of Nitrogen Fertilizer Combined with Humic Acid on Soil Bacterial Communities

Studies have indicated that both nitrogen and humic acid fertilizers directly stimulate microbial population growth by supplying nutrients [43,44,45,46]. This leads to an increase in microbial abundance, enhanced microbial activity, and a shift in microbial diversity. High soil microbial diversity is essential for agricultural ecosystem productivity and stability [47], and SOC, AK, and TN significantly impact soil bacteria [38]. Research on rhizosphere bacterial communities has shown that pH, SOC, and AP are the most critical factors influencing soil bacterial communities [48]. In this experiment, humic acid treatment resulted in higher soil nutrient indices than nitrogen fertilizer application alone or the control treatment. Compared to the control group, the application of nitrogen fertilizer reduced microbial diversity, consistent with the findings of Sun and Huang [49,50]. Humic acid stimulates microbial diversity and influences microbial community structure by altering soil physicochemical properties and root exudate composition [31]. Soil bacterial communities play a vital role in carbon and nitrogen conversion, nutrient cycling, and maintaining soil structure, and serve as indicators of soil nutrients [51]. Soil pH affects microbial activity and nutrient availability. Humic acid helps neutralize soil pH, creating a more favorable environment for bacterial growth [52]. In this study, Proteobacteria, Actinomycetes, Chloroflexi, and Acidobacteria comprised over 70% of the total bacterial community, which is consistent with the typical trends observed in agricultural soils [53]. In the treatment combining nitrogen fertilizer and humic acid, the phyla Proteobacteria and Actinomycetes were stimulated, whereas the abundance of Acidobacteria decreased. Proteobacteria play a key role in the nitrogen cycle, enhancing nitrogen utilization and increasing phosphorus availability [54,55]. The Actinomycetes phylum can secrete antibiotics and enzymes to inhibit pathogenic bacterial growth and promote organic matter decomposition and nutrient release. The Bacteroidetes and Bacillus phyla degrade carbohydrates and enhance nutrient cycling [56]. The phylum Planctomycetes contains nitrogen-cycling bacteria with nitrogen-fixing ability. However, humic acid gives Proteobacteria a competitive advantage in nitrite competition, thereby inhibiting the growth of Planctomycetes [57,58,59]. The phylum Planctomycetes contains nitrogen-cycling bacteria with nitrogen-fixing ability. However, humic acid gives Proteobacteria a competitive advantage in nitrite competition, inhibiting the growth of Planctomycetes [60,61]. No valid sequences from the phylum Planctomycetes were detected. This does not imply the complete absence of this group in the soil but rather indicates its extremely low abundance within the quinoa rhizosphere microbiome, falling below the detection limit of the sequencing method employed in this experiment. The Illumina MiSeq platform was used for 16S rRNA gene sequencing. Based on data quality control standards (filtered sequence quality score Q30 > 95%, operational taxonomic unit (OTU) threshold 97%), the detection limit was approximately 0.01% of the total community abundance. Combined with the soil characteristics of the experimental site and rhizosphere effect analysis, the core reason for the undetection of Planctomycetes lies in the fact that quinoa rhizosphere secretions, primarily composed of organic acids and amino acids, may negatively regulate the chemotaxis and colonization of Planctomycetes in the rhizosphere. This results in a competitive disadvantage in the rhizosphere microenvironment compared to dominant groups such as Proteobacteria and Actinobacteria, further reducing the probability of their detection. Although no valid sequences for this group were detected, its functional characteristics (involvement in ammonia oxidation) and soil nitrogen transformation results (ammonium nitrogen content increased by 15.6% under N_2_H treatment) suggest that other functional groups (e.g., ammonia-oxidizing bacteria within the Proteobacteria phylum) may have compensated for its ecological function, ensuring the integrity of the rhizosphere nitrogen cycling process. Studies have shown that combining humic acid with nitrogen fertilizer significantly impacts soil bacterial community structure, boosts organic matter decomposition and nutrient cycling, and improves nutrient availability for plant growth [23,62].

### 4.3. Production Analysis

Fertilization treatments significantly boost crop yields by improving the soil structure and nutrient availability. Humic acid fertilizers offer dual benefits: (1) significant soil condition improvements and (2) a yield increase of 7.88–15.51% (*p* < 0.05) with a single application (Figure 5), confirming its intrinsic yield-enhancing capacity. The combined application of nitrogen fertilizer and humic acid outperformed the single nitrogen application, with yield increases ranging from 23.53% to 69.65% (*p* < 0.01) (Table 2). This suggests that humic acid promotes nitrogen utilization efficiency through multiple mechanisms: enhancing nitrogen retention in the root zone and improving soil microporosity to boost nutrient accessibility and crop yield [63]. Optimization of rhizosphere microenvironment to promote nitrogen acquisition. Humic acid improves the physical and chemical properties of rhizosphere soil, creating a favorable environment for root growth and nitrogen absorption. In this study, N2H treatment increased soil organic carbon (SOC) by 33.31% and available potassium (AK) by 20.23% compared to the N2 treatment. Under medium nitrogen conditions (150 kg ha^−1^), the combined application of humic acid achieved the highest yield (a 69.65% increase compared to the control), identifying the optimal fertilizer ratio for quinoa cultivation. The yield sequence was N2H > N3H > N1H (*p* < 0.05). When the nitrogen content exceeds moderate levels, the yields begin to decline. The increase in core microbial species driven by humic acid may enhance microbial cooperation, improving plant health and yield [64], consistent with our findings. At high nitrogen levels, quinoa yield decreases, likely due to soil salinity accumulation, which damages the soil structure and negatively impacts nutrient absorption and plant growth.

## 5. Conclusions

This two-year field trial quantified the effects of four nitrogen fertilizer rates (N0: 0, N1: 120, N2: 150, N3: 180 kg ha^−1^) combined with humic acid (HA: 1500 kg ha^−1^) on the root-zone ecosystem and yield of quinoa. Results indicated that humic acid application significantly enhanced soil nutrient supply capacity: under optimal conditions, soil organic carbon (SOC), total nitrogen (TN), available phosphorus (AP), and available potassium (AK) contents all increased. Concurrently, HA application stimulated soil microbial activity, markedly increasing quinoa yield. Humic acid altered rhizosphere bacterial community composition and diversity by elevating the relative abundance of beneficial phyla (e.g., Proteobacteria and Actinobacteria) while reducing that of the Flexneria phylum. Notably, the positive effects of HA—including enhanced soil nutrient content and bacterial diversity—were pronounced at low-to-medium nitrogen levels. However, these benefits diminished under high nitrogen application (180 kg ha^−1^ N), where excessive nitrogen input appeared to counteract HA’s stimulatory effects.

The differential efficacy of humic acid across nitrogen fertilizer levels indicates that future quinoa cultivation management should move beyond uniform nitrogen or humic acid application towards precision fertilization strategies. The findings indicate that the combination of medium nitrogen fertilizer (150 kg ha^−1^) with humic acid (1500 kg ha^−1^) constitutes the optimal fertilization regimen, providing theoretical support for rational nitrogen and humic acid management in quinoa production. Whilst this study offers valuable insights into the effects of combined nitrogen-humic acid application on quinoa rhizosphere ecosystems and yield, these findings appear confined to experimental field conditions. For broader implementation, multi-regional field trials, long-term monitoring of soil ecological processes, and in-depth investigation of rhizosphere microbial-nutrient interactions may be required to better understand the long-term effects of this fertilization strategy across diverse agroecological zones.

## Figures and Tables

**Table 1:** Specific Fertilization Conditions for Each Treatment Group.

Treatment Code	N Fertilizer (kg ha^−1^)	H Fertilizer (kg ha^−1^)
CK	0	0
N1	120	0
N2	150	0
N3	180	0
H	0	1500
N1H	120	1500
N2H	150	1500
N3H	180	1500

**Table 2:** Basic properties of humic acid fertilizer.

Humic Acid Fertilizer	pH	Humic Acid (%)	Fulvic Acid (%)	Organic Matter (%)	Moisture Content (%)
HA	9.2	40	35	50	10

**Table 3:** Quinoa Grain Yield and Its Component Factors Under Various Fertilizer Treatments in 2023 and 2024.

	Process	Number of Spikes per Plant	1000-Grain Weight (g)	Grain Weight per Plant (g)	Main Spike Length (cm)	Yield (kg ha^−1^)
2 0 2 3	CK	17.7 ± 0.67 c	3.37 ± 0.14 b	38.21 ± 0.89 c	19.44 ± 0.86 c	2636.49 ± 123.01 c
	N1	20.7 ± 0.33 ab	3.68 ± 0.15 ab	53.57 ± 1.38 a	30.21 ± 0.52 a	3696.33 ± 108.18 a
	N2	21.3 ± 0.67 a	4.26 ± 0.13 a	47.46 ± 1.26 b	28.56 ± 0.57 ab	3275.02 ± 123.22 ab
	N3	19.3 ± 0.67 b	4.18 ± 0.07 a	44.98 ± 1.40 b	25.41 ± 0.91 b	3103.62 ± 104.53 bc
	H	18.3 ± 0.33 c	4.12 ± 0.10 b	41.22 ± 1.41 d	22.53 ± 0.68 b	2844.18 ± 117.06 d
	N1H	23.7 ± 0.67 ab	4.41 ± 0.11 ab	51.93 ± 1.05 c	24.11 ± 1.04 b	3583.10 ± 124.25 c
	N2H	25.3 ± 0.33 a	4.32 ± 0.10 ab	64.82 ± 1.57 a	34.98 ± 1.15 a	4472.86 ± 130.76 a
	N3H	22.3 ± 0.67 b	4.64 ± 0.14 a	57.56 ± 1.31 b	32.67 ± 0.59 a	3971.78 ± 138.50 b
	N	**	**	**	**	**
	H	**	**	**	**	**
	N × H	NS	NS	**	**	**
2 0 2 4	CK	14.5 ± 0.50 c	3.38 ± 0.13 b	37.43 ± 0.11 c	29.67 ± 0.63 c	2582.41 ± 118.93 c
	N1	18.5 ± 0.50 b	3.40 ± 0.05 b	44.54 ± 0.76 b	39.58 ± 0.65 a	3073.55 ± 124.46 b
	N2	22.5 ± 0.50 a	4.29 ± 0.16 a	50.52 ± 0.55 a	36.78 ± 0.79 ab	3485.83 ± 127.68 a
	N3	21.7 ± 0.67 a	4.43 ± 0.22 a	50.06 ± 0.54 a	34.06 ± 0.56 b	3454.22 ± 102.98 a
	H	16.5 ± 0.50 c	3.88 ± 0.06 b	43.23 ± 0.38 d	32.69 ± 0.69 c	2983.02 ± 115.90 c
	N1H	20.5 ± 0.50 a	3.63 ± 0.03 c	51.81 ± 0.67 c	37.80 ± 0.35 b	3574.63 ± 115.78 b
	N2H	20.7 ± 0.33 b	4.58 ± 0.10 a	62.14 ± 0.89 a	37.63 ± 0.40 b	4287.61 ± 104.50 a
	N3H	24.5 ± 0.50 a	3.86 ± 0.02 b	46.64 ± 0.61 b	42.45 ± 0.81 a	3218.11 ± 114.83 bc
	N	**	**	**	**	**
	H	**	NS	**	**	**
	N × H	**	**	**	**	*

**Note:** CK (0 kg ha^−1^ nitrogen fertilizer); N1 (120 kg ha^−1^ nitrogen fertilizer); N2 (150 kg ha^−1^ nitrogen fertilizer); N3 (180 kg ha^−1^ nitrogen fertilizer); H (1500 kg ha^−1^ humic acid); N1H (120 kg ha^−1^ nitrogen fertilizer and 1500 kg ha^−1^ humic acid); N2H (150 kg ha^−1^ nitrogen fertilizer and 1500 kg ha^−1^ humic acid); and N3H (180 kg ha^−1^ nitrogen fertilizer and 1500 kg ha^−1^ humic acid). Different letters within the same column indicate statistically significant differences according to Duncan’s multiple range test (*p* ≤ 0.05). N, H, and N × H represent the nitrogen fertilizer level, humic acid level, and their interaction, respectively. * indicates a significant effect (*p* < 0.05), ** indicates a highly significant effect (*p* < 0.01), and NS indicates no significant difference.

**Table 4:** Relationships between the response ratio of the soil microbial community and environmental factors, as well as soil physicochemical properties (only significant relationships are presented).

Variables	Soil Nutrients	Linear Equation	*p*-Value
Bacterial OTUs	pH	Y = 0.4724 × X − 0.01841	0.002
Bacterial OTUs	AK	Y = −0.06903 × X − 0.00388	0.002
Bacterial OTUs	TN	Y = −0.05174 × X + 0.03143	0.002
Bacterial Ace index	pH	Y = −0.11769 × X − 0.01686	0.009
Bacterial Ace index	AP	Y = −0.00765 × X − 0.01618	0.003
Bacterial Ace index	AK	Y = −0.05912 × X − 0.00594	<0.001
Bacterial Ace index	SOC	Y = −0.0958 × X − 0.00355	0.011
Bacterial Ace index	TN	Y = −0.2963 × X + 0.0099	0.003
Bacterial Chao index	pH	Y = 0.21113 × X − 0.01515	0.002
Bacterial Chao index	AP	Y = −0.2838 × X − 0.00822	0.010
Bacterial Chao index	AK	Y = −0.06512 × X − 0.00472	<0.001
Bacterial Chao index	SOC	Y = −0.10215 × X − 0.00264	0.005
Bacterial Chao index	TN	Y = −0.03963 × X + 0.01959	0.004
Bacterial Shannon index	pH	Y = −0.00658 × X − 0.01349	0.007
Bacterial Shannon index	AK	Y = −0.02152 × X − 0.00855	<0.001
Bacterial Shannon index	TN	Y = −0.01173 × X − 0.00186	0.009

## Data Availability

All data generated or analyzed during this study are included in this published article.

## Supplementary Materials

The following supporting information can be downloaded at: https://www.mdpi.com/article/10.3390/plants14243850/s1. Table S1: Analysis of variance for soil physical and chemical properties under different fertilization treatments in 2023; Table S2: Analysis of variance for soil physical and chemical properties under different fertilization treatments in 2024.

## References

[1] Tilman D., Cassman K.G., Matson P.A., Naylor R., Polasky S. (2002). Agricultural sustainability and intensive production practices. Nature.

[2] Ladha J.K., Reddy C.K., Padre A.T., van Kessel C. (2011). Role of Nitrogen Fertilization in Sustaining Organic Matter in Cultivated Soils. J. Environ. Qual..

[3] Pan G., Smith P., Pan W. (2009). The role of soil organic matter in maintaining the productivity and yield stability of cereals in China. Agric. Ecosyst. Environ..

[4] Williams C.L., Liebman M., Edwards J.W., James D.E., Singer J.W., Arritt R., Herzmann D. (2008). Patterns of Regional Yield Stability in Association with Regional Environmental Characteristics. Crop Sci..

[5] Liu J., You L., Amini M., Obersteiner M., Herrero M., Zehnder A.J.B., Yang H. (2010). A high-resolution assessment on global nitrogen flows in cropland. Proc. Natl. Acad. Sci. USA.

[6] Cassman K.G., Dobermann A., Walters D.T. (2002). Agroecosystems, nitrogen-use efficiency, and nitrogen management. Ambio.

[7] Robertson G.P., Vitousek P.M. (2009). Nitrogen in Agriculture: Balancing the Cost of an Essential Resource. Annu. Rev. Environ. Resour..

[8] Lassaletta L., Billen G., Grizzetti B., Anglade J., Garnier J. (2014). 50 year trends in nitrogen use efficiency of world cropping systems: The relationship between yield and nitrogen input to cropland. Environ. Res. Lett..

[9] Van Drecht G., Bouwman A.F., Knoop J.M., Beusen A.H.W., Meinardi C.R. (2003). Global modeling of the fate of nitrogen from point and nonpoint sources in soils, groundwater, and surface water. Glob. Biogeochem. Cycles.

[10] Galloway J.N., Townsend A.R., Erisman J.W., Bekunda M., Cai Z., Freney J.R., Martinelli L.A., Seitzinger S.P., Sutton M.A. (2008). Transformation of the Nitrogen Cycle: Recent Trends, Questions, and Potential Solutions. Science.

[11] Melero S., Vanderlinden K., Ruiz J.C., Madejon E. (2008). Long-term effect on soil biochemical status of a Vertisol under conservation tillage system in semi-arid Mediterranean conditions. Eur. J. Soil Biol..

[12] Liu L., Yao S., Zhang H., Muhammed A., Xu J., Li R., Zhang D., Zhang S., Yang X. (2019). Soil nitrate nitrogen buffer capacity and environmentally safe nitrogen rate for winter wheat-summer maize cropping in Northern China. Agric. Water Manag..

[13] Dong N.-Q., Lin H.-X. (2020). Higher yield with less nitrogen fertilizer. Nat. Plants.

[14] Li T., Liang Y., Wang Z., Zhang W., Wang L., Zhou Q., Xu W. (2018). Tissue-engineered scaffold based on carboxymethyl chitin or chitosan for corneal epithelial transplantation. Polym. J..

[15] Xu Y., Li M., Ding H., Ma Y., Yang Y., Feng L. (2025). Comparative effects of humic acid biostimulation on soil properties, growth, and fragrance of Rosa rugosa. Ind. Crops Prod..

[16] Gerke J. (2018). Concepts and Misconceptions of Humic Substances as the Stable Part of Soil Organic Matter: A Review. Agronomy.

[17] Chen X., Kou M., Tang Z., Zhang A., Li H., Wei M. (2017). Responses of root physiological characteristics and yield of sweet potato to humic acid urea fertilizer. PLoS ONE.

[18] Suman S., Spehia R.S., Sharma V. (2016). Humic acid improved efficiency of fertigation and productivity of tomato. J. Plant Nutr..

[19] Selladurai R., Purakayastha T.J. (2015). Effect of humic acid multinutrient fertilizers on yield and nutrient use efficiency of potato. J. Plant Nutr..

[20] Khan R.U., Khan M.Z., Khan A., Saba S., Hussain F., Jan I.U. (2017). Effect of Humic Acid on Growth and Crop Nutrients Status of Wheat on Two Different Soil. J. Plant Nutr..

[21] Ahmad T., Khan R., Nawaz Khattak T. (2018). Effect of humic acid and fulvic acid based liquid and foliar fertilizers on the yield of wheat crop. J. Plant Nutr..

[22] Rose M.T., Patti A.F., Little K.R., Brown A.L., Jackson W.R., Cavagnaro T.R., Sparks D.L. (2014). Chapter Two—A Meta-Analysis and Review of Plant-Growth Response to Humic Substances: Practical Implications for Agriculture. Advances in Agronomy.

[23] Pukalchik M., Kydralieva K., Yakimenko O., Fedoseeva E., Terekhova V. (2019). Outlining the Potential Role of Humic Products in Modifying Biological Properties of the Soil—A Review. Front. Environ. Sci..

[24] Hu Y.W., Li Q.K., Song C.J., Jin X.H. (2021). EFFECT OF HUMIC ACID COMBINED WITH FERTILIZER ON THE IMPROVEMENT OF SALINE-ALKALI LAND AND COTTON GROWTH. Appl. Ecol. Environ. Res..

[25] Li Y. (2020). Research Progress of Humic Acid Fertilizer on the Soil. J. Phys. Conf. Ser..

[26] Amoah-Antwi C., Kwiatkowska-Malina J., Szara E., Fenton O., Thornton S.F., Malina G. (2022). Assessing Factors Controlling Structural Changes of Humic Acids in Soils Amended with Organic Materials to Improve Soil Functionality. Agronomy.

[27] Ullah S., He P., Ai C., Zhao S., Ding W., Song D., Zhang J., Huang S., Abbas T., Zhou W. (2020). How Do Soil Bacterial Diversity and Community Composition Respond under Recommended and Conventional Nitrogen Fertilization Regimes?. Microorganisms.

[28] Wu M., Song M., Liu M., Jiang C., Li Z. (2016). Fungicidal activities of soil humic/fulvic acids as related to their chemical structures in greenhouse vegetable fields with cultivation chronosequence. Sci. Rep..

[29] Dawood M.G., Abdel-Baky Y.R., El-Awadi M.E.-S., Bakhoum G.S. (2019). Enhancement quality and quantity of faba bean plants grown under sandy soil conditions by nicotinamide and/or humic acid application. Bull. Natl. Res. Cent..

[30] García A.C., de Souza L.G.A., Pereira M.G., Castro R.N., García-Mina J.M., Zonta E., Lisboa F.J.G., Berbara R.L.L. (2016). Structure-Property-Function Relationship in Humic Substances to Explain the Biological Activity in Plants. Sci. Rep..

[31] Lumactud R.A., Gorim L.Y., Thilakarathna M.S. (2022). Impacts of humic-based products on the microbial community structure and functions toward sustainable agriculture. Front. Sustain. Food Syst..

[32] Li Y., Fang F., Wei J., Wu X., Cui R., Li G., Zheng F., Tan D. (2019). Humic Acid Fertilizer Improved Soil Properties and Soil Microbial Diversity of Continuous Cropping Peanut: A Three-Year Experiment. Sci. Rep..

[33] Rajkumar M., Ae N., Prasad M.N.V., Freitas H. (2010). Potential of siderophore-producing bacteria for improving heavy metal phytoextraction. Trends Biotechnol..

[34] Nardi S., Pizzeghello D., Muscolo A., Vianello A. (2002). Physiological effects of humic substances on higher plants. Soil Biol. Biochem..

[35] Hedges L.V., Gurevitch J., Curtis P.S. (1999). THE META-ANALYSIS OF RESPONSE RATIOS IN EXPERIMENTAL ECOLOGY. Ecology.

[36] Pawlowski J., Kelly-Quinn M., Altermatt F., Apothéloz-Perret-Gentil L., Beja P., Boggero A., Borja A., Bouchez A., Cordier T., Domaizon I. (2018). The future of biotic indices in the ecogenomic era: Integrating (e)DNA metabarcoding in biological assessment of aquatic ecosystems. Sci. Total Environ..

[37] Hui D., Porter W., Phillips J.R., Aidar M.P.M., Lebreux S.J., Schadt C.W., Mayes M.A. (2020). Phosphorus rather than nitrogen enhances CO2 emissions in tropical forest soils: Evidence from a laboratory incubation study. Eur. J. Soil Sci..

[38] Geisseler D., Scow K.M. (2014). Long-term effects of mineral fertilizers on soil microorganisms—A review. Soil Biol. Biochem..

[39] Liu L., Greaver T.L. (2010). A global perspective on belowground carbon dynamics under nitrogen enrichment. Ecol. Lett..

[40] Guo Y., Ma Z., Ren B., Zhao B., Liu P., Zhang J. (2022). Effects of Humic Acid Added to Controlled-Release Fertilizer on Summer Maize Yield, Nitrogen Use Efficiency and Greenhouse Gas Emission. Agriculture.

[41] Ai C., Zhang S., Zhang X., Guo D., Zhou W., Huang S. (2018). Distinct responses of soil bacterial and fungal communities to changes in fertilization regime and crop rotation. Geoderma.

[42] Yue Y., Fan Y., Zhuqing L., Kui C. (2024). Artificial humic acid improves P availability via regulating P-cycling microbial communities for crop growth. Plant Soil.

[43] Chu H., Lin X., Fujii T., Morimoto S., Yagi K., Hu J., Zhang J. (2007). Soil microbial biomass, dehydrogenase activity, bacterial community structure in response to long-term fertilizer management. Soil Biol. Biochem..

[44] Joergensen R.G., Mäder P., Fließbach A. (2010). Long-term effects of organic farming on fungal and bacterial residues in relation to microbial energy metabolism. Biol. Fertil. Soils.

[45] Murugan R., Kumar S. (2013). Influence of long-term fertilisation and crop rotation on changes in fungal and bacterial residues in a tropical rice-field soil. Biol. Fertil. Soils.

[46] Tao R., Liang Y., Wakelin S.A., Chu G. (2015). Supplementing chemical fertilizer with an organic component increases soil biological function and quality. Appl. Soil Ecol..

[47] Francioli D., Schulz E., Lentendu G., Wubet T., Buscot F., Reitz T. (2016). Mineral vs. Organic Amendments: Microbial Community Structure, Activity and Abundance of Agriculturally Relevant Microbes Are Driven by Long-Term Fertilization Strategies. Front. Microbiol..

[48] Wang J., Rhodes G., Huang Q., Shen Q. (2018). Plant growth stages and fertilization regimes drive soil fungal community compositions in a wheat-rice rotation system. Biol. Fertil. Soils.

[49] Sun Y.-F., Shen J.-P., Zhang C.-J., Zhang L.-M., Bai W.-M., Fang Y., He J.-Z. (2017). Responses of soil microbial community to nitrogen fertilizer and precipitation regimes in a semi-arid steppe. J. Soils Sediments.

[50] Huang X., Liu Y., Li Y., Guo P., Fang X., Yi Z. (2018). Foliage application of nitrogen has less influence on soil microbial biomass and community composition than soil application of nitrogen. J. Soils Sediments.

[51] Tosi M., Deen W., Drijber R., McPherson M., Stengel A., Dunfield K. (2021). Long-term N inputs shape microbial communities more strongly than current-year inputs in soils under 10-year continuous corn cropping. Soil Biol. Biochem..

[52] Fernández-Calviño D., Bååth E. (2010). Growth response of the bacterial community to pH in soils differing in pH. FEMS Microbiol. Ecol..

[53] Li F., Chen L., Zhang J., Yin J., Huang S. (2017). Bacterial Community Structure after Long-term Organic and Inorganic Fertilization Reveals Important Associations between Soil Nutrients and Specific Taxa Involved in Nutrient Transformations. Front. Microbiol..

[54] Zhang L., Peng Y., Zhou J., George T.S., Feng G. (2020). Addition of fructose to the maize hyphosphere increases phosphatase activity by changing bacterial community structure. Soil Biol. Biochem..

[55] Wei X., Hu Y., Razavi B.S., Zhou J., Shen J., Nannipieri P., Wu J., Ge T. (2019). Rare taxa of alkaline phosphomonoesterase-harboring microorganisms mediate soil phosphorus mineralization. Soil Biol. Biochem..

[56] Partanen P., Hultman J., Paulin L., Auvinen P., Romantschuk M. (2010). Bacterial diversity at different stages of the composting process. BMC Microbiol..

[57] Lu J., Zhang Y., Wu J., Wang J. (2020). Nitrogen removal in recirculating aquaculture water with high dissolved oxygen conditions using the simultaneous partial nitrification, anammox and denitrification system. Bioresour. Technol..

[58] Xu J.J., Zhu X.L., Zhang Q.Q., Cheng Y.F., Xu L.Z.J., Zhu Y.H., Ji Z.Q., Jin R.C. (2018). Roles of MnO_2_ on performance, sludge characteristics and microbial community in anammox system. Sci. Total Environ..

[59] Pijuan M., Ribera-Guardia A., Balcázar J.L., Micó M.M., de la Torre T. (2020). Effect of COD on mainstream anammox: Evaluation of process performance, granule morphology and nitrous oxide production. Sci. Total Environ..

[60] Malam Issa O., Défarge C., Trichet J., Valentin C., Rajot J.L. (2009). Microbiotic soil crusts in the Sahel of Western Niger and their influence on soil porosity and water dynamics. CATENA.

[61] Tiwari O.N., Bhunia B., Mondal A., Gopikrishna K., Indrama T. (2018). System metabolic engineering of exopolysaccharide-producing cyanobacteria in soil rehabilitation by inducing the formation of biological soil crusts: A review. J. Clean. Prod..

[62] Wang J.L., Liu K.L., Zhao X.Q., Gao G.-F., Wu Y.H., Shen R.F. (2021). Microbial keystone taxa drive crop productivity through shifting aboveground-belowground mineral element flows. Sci. Total Environ..

[63] Mosaad I.S.M., Serag A.H.I., Sheta M.H. (2022). Promote sugar beet cultivation in saline soil by applying humic substances in-soil and mineral nitrogen fertilization. J. Plant Nutr..

[64] Qiao Y., Wang T., Huang Q., Guo H., Zhang H., Xu Q., Shen Q., Ling N. (2023). Core species impact plant health by enhancing soil microbial cooperation and network complexity during community coalescence. Soil Biol. Biochem..

