# Supplementary material for: Humic Acid Enhances Soil Fertility and Microbial Diversity Under Optimized Nitrogen Fertilization in Quinoa Rhizosphere
Source: Plants (Basel). 2025 Dec 17;14(24):3850. doi: 10.3390/plants14243850 (PMC12736530; doi:10.3390/plants14243850)
Supplement: Supplementary file 1 [file plants-14-03850-s001.zip › plants-3967813-supplementary.pdf]

Table S1 Analysis of variance for soil physical and chemical properties under different fertilization treatments in 2023

Variance source	Total nitrogen	Organic carbon	C/N	pH	Available P	Available K
N	**	*	**	*	**	**
H	**	**	**	**	**	*
N×H	*	NS	**	**	*	NS

Table S2 Analysis of variance for soil physical and chemical properties under different fertilization treatments in 2024

Variance source	Total nitrogen	Organic carbon	C/N	pH	Available P	Available K
N	**	**	**	NS	**	*
H	**	**	**	**	**	**
N×H	**	NS	*	*	*	NS

Note:N、H and N × H respectively represent nitrogen level, humic acid level, and their interaction, * indicating a significant effect ($P < 0.05$), and ** indications a highly significant effect ($P < 0.01$), ns represents no significant difference.
